# A long term resilient modulus rate dependent model for coarse fine mixtures geomaterials under freezing and thawing cyclic

**DOI:** 10.1038/s41598-022-19647-x

**Published:** 2022-10-19

**Authors:** Ke Wang, Liang Tang, Shuang Tian, XianZhang Ling, Yangsheng Ye, Degou Cai, Min Liu

**Affiliations:** 1 School of Civil Engineering, Harbin Institute of Technology, Harbin, 150090 Heilongjiang China; 2 Heilongjiang Research Center for Rail Transit Engineering in Cold Regions, Harbin, 150090 Heilongjiang China; 3 Railway Engineering Research Institute, China Academy of Railway Sciences Corporation Limited, Beijing, 100081 China

**Keywords:** Civil engineering, Climate change

## Abstract

The cyclic loading frequency (*f*_cyc_) effects on the resilient modulus (*M*_r_) of freezing–thawing coarse–fine mixtures geomaterials (FTCFG) have always been a research hotspot. A series of long-term cyclic triaxial tests were conducted on FTCFG having different fines content (FC) under different number of freeze–thaw cycles (*N*_FT_) to investigate the effect of *f*_cyc_ and deviator stress amplitude (*q*_cyc_) on the *M*_r_ of FTCFG. The freezing–thawing cyclic was found to improve the *M*_r_ of FTCFG. Additionally, *M*_r_ of FTCFG shown an obviously rate-dependent characteristics. Then three kinetic effects (rate effect, piston effect, and fatigue effect) are discussed in systemically which are related to *q*_cyc_, *f*_cyc_ and moisture holding capacity (*w*_h_). Finally, a rate dependent model of long-term resilient modulus was developed to predict FTCFG materials’ resilient moduli as a function of *q*_cyc_, *f*_cyc_ and *w*_h_. The comparisons between the calculation and experimental results reveal that the present model describes the *M*_r_ of FTCFG well.

## Introduction

Subgrade fillers having different content of fine matrix form the substructure of the high-speed rail. The coupling effect of freezing–thawing cycle and cyclic loading on the dynamic characteristics of coarse–fine mixtures geomaterials is a key factor which severely restricts the running speed of high-speed railway in cold regions. The resilient modulus (*M*_r_), defined by Seed et al. as the ratio of repeated deviator stress to axial recoverable strain in a cyclic triaxial test (*M*_r_ = *q*_cyc_/${\upvarepsilon}_{\text{r}}$), is widely used as a stiffness parameter in pavement engineering to determine soil deformation under cyclic traffic loads^1–8^.

Currently, there are two different effects of number of freeze–thaw cycles (*N*_FT_) on the ${\text{M}}_{\text{r}}$ of FTCFG. On the one hand, freezing–thawing cycles weaken the *M*_r_ of FTCFG, and the degree of influence mainly depends on the moisture content of FTCFG, fine matrix content and compactness of the coarse grain. With the increase of *N*_FT_, the *M*_r_ of FTCFG tend to stabilize after the test specimen undergoes 10 freezing–thawing cycles. When the fine particle content is less than 1%, the freezing–thawing cycle has no significant effect on coarse–fine mixtures^9–16^. On the other hand, it was found that the freezing–thawing cycle has no negative effect on the *M*_r_ of FTCFG^17,18^. It was found that the *M*_r_ of FTCFG increases after a freezing–thawing cycle. Moreover, although the strength of coarse–fine mixtures would decrease significantly after freezing and thawing, part of the water would be discharged, and the strength of coarse–fine mixtures would recover during thawing^19^. The effect of low frequency (*f*_cyc_ ≤ 10 Hz) on the *M*_r_ was negligible in studies where the coarse–fine mixtures did not undergo freezing–thawing cycles^20–23^. But the effect of high frequency (*f*_cyc_ > 10 Hz) on the *M*_r_ was not negligible^24–27^. Furthermore, a clear influence was observed on the *M*_r_ of freezing–thawing geomaterials in studies where a low frequency range was simulated, that is, 0.5 Hz ≤ *f*_cyc_ ≤ 3 Hz^15^. In addition, a proven conclusion is that the fines contents significantly influenced the *M*_r_ of coarse–fine mixtures that did not undergo freezing–thawing cycles^6–8,28–30^. Although studies on the effects of freezing–thawing cycles on the *M*_r_ of coarse–fine mixtures are really mature^15,31–33^, the *M*_r_ of FTCFG under high frequency are yet unknown.

Based on a large number of experimental data and phenomena, a series of phenomenological models of *M*_r_ are proposed. Uzan^34^ proposed a model to present the relationship between *M*_r_ and confining pressure and cyclic deviator stress, whereas it does not consider the effect of *f*_cyc_ on *M*_r_, as it ignores geomaterials suction. It can be modified only by adopting two stress state variables. Ng and Yung^35^ proposed a semi-empirical equation for shear modulus, G_0_, of unsaturated geomaterials. The semi-empirical incorporates net confining pressure and matric suction. To completely describe *M*_r_ of unsaturated geomaterials, a model is proposed by employing the advantages of each equation^36^. It can describe the influence of net stress, cyclic deviator stress and suction on *M*_r_ of unsaturated geomaterials. The rate dependent properties of materials have been widely studied in the fields of concrete and crystalline metallic materials^37,38^. Since that the FTCFG material is unbound geomaterials, it is very difficult to control the test under freezing–thawing cyclic and high cyclic loading frequency. Hence, the effect of cyclic loading frequency on *M*_r_ of unbound geomaterials has been poorly considered in previous phenomenological model, especially for the FTCFG with different fine content (FC) and suffer long-term cyclic loading.

The purpose of this paper is to put forward a rate dependent prediction model of *M*_r_ that is suitable for FTCFG materials under high cyclic loading frequency. Firstly, four types of specimen were fabricated by mixing different FC (6% ≤ FC ≤ 21%) and coarse particles and undergoing the freezing–thawing cycle (*N*_FT_ = 10). A series of long-term dynamic triaxial loading tests (5 Hz ≤ *f*_cyc_ ≤ 20 Hz, 50 kPa ≤ *q*_cyc_ ≤ 125 kPa) were conducted using the aforementioned specimens. Then, the effects of cyclic loading frequency on the *M*_r_ of FTCFG with different fine contents were systematically analyzed and discussed. Finally, a rate dependent model of long-term resilient modulus was developed to predict FTCFG materials’ resilient moduli as a function of *q*_cyc_, *f*_cyc_ and *w*_h_.

## Materials and methodology

### Material properties

The materials of the test specimens were obtained from the subgrade of the Harbin–Dalian high-speed railway after operation. The maximum fine matrix content of the upper layer of the subgrade was found to be approximately 21.1%^39^. Therefore, the FC of present test were set as 6%, 11%, 16%, and 21%. FC_2_ is the content of fines less than 2 mm, which is used to calculate the water-holding moisture content (*w*_h_). Specimen with different FC shown different *w*_h_, different optimal water content (*w*_opt_), and different dry density (${\uprho}_{\text{d}}$). All the specimens (Diameter = 100 mm, Height = 200 mm) were prepared with the same total water content (*w* = 6.19%). The liquid-plastic limit test is only carried out for the clay with the particle size less than 0.5 mm. When the particle composition of soil is different, the measured liquid plastic limit index is also different. When the fine particle content of sample was 6%, LL was 18.8%. When the fine particle content of sample was 21%, LL was 25.0%. According to the Unified Soil Classification System, the tested samples are poorly graded sand-gravel soil, which is classified as Type B (base course material in China’s HSR)^40^. Also, the tested materials can be classified as poorly graded gravel with clay and sand as per the Unified Soil Classification System (USCS) ASTM D2487^41^. Table 1 lists the details of the experimental materials. Further details regarding the specimen preparation were displayed in our early work^42^.

**Table 1:** Summary of materials.

Features	Information			
*w*, %	6.19			
FC, %	6	11	16	21
FC_2_, %	27.79	32.10	35.81	39.40
*w*_h_, %	21.01	19.31	17.35	15.71
*w*_opt_, %	5.94	6.19	7.51	8.49
${\uprho}_{\text{d}}$, Mg/m^3^	1.798	1.895	1.956	2.012
${\uprho}_{\text{dmax}}$, Mg/m^3^	1.997	2.105	2.173	2.235

**Figure 1:**
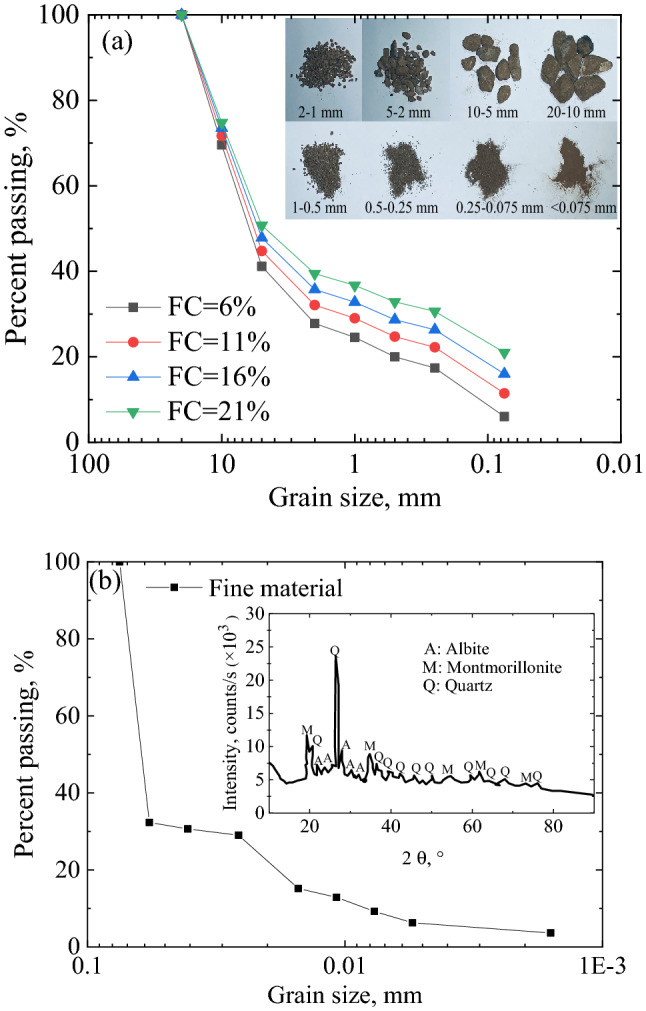
Grain size distribution of the samples: (**a**) focusing on the grain size more than 0.075 mm and (**b**) focusing on fines matrix and showing the XRD test results.

Figure 1 shows the gradation curve of the specimens and the results of XRD test. According to previous research, the specimen would attain a stable state after undergoing 6 freezing–thawing cycles^14^. Therefore, we focused on the specimens that underwent 10 freezing–thawing cycles (Guarantee state stability) at present test. Further details regarding the freezing–thawing cycle of the specimen are stated in Tian^14^.

### Test equipment

**Figure 2:**
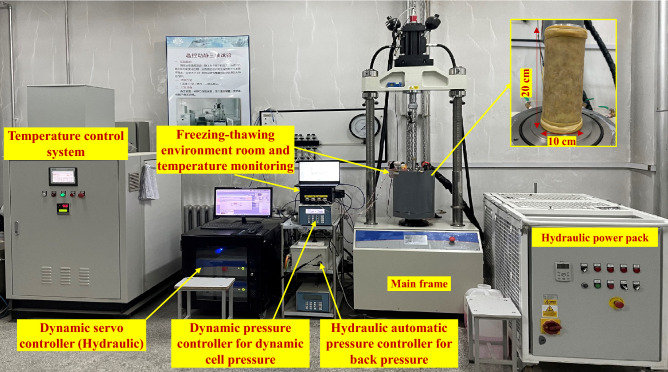
Test equipment.

The tests were conducted using the temperature controlled dynamic and static triaxial equipment of Harbin Institute of Technology’s college of civil engineering, as illustrated in Fig. 2. The major technical parameters of the test equipment are as follows: the maximum axial force is 50 kN; the maximum confining pressure is 2 MPa; the operating temperature is − 30 to 30 °C; the axial and circumferential extensometer resolutions are ± 4 mm and − 2.5 to 8 mm, respectively; and the measurement accuracy is 0.5%. The sample size is 100 mm in diameter and 200 mm in height. Further details on the cyclic triaxial test were displayed in our early work^14^.

### Cyclic triaxial tests

**Figure 3:**
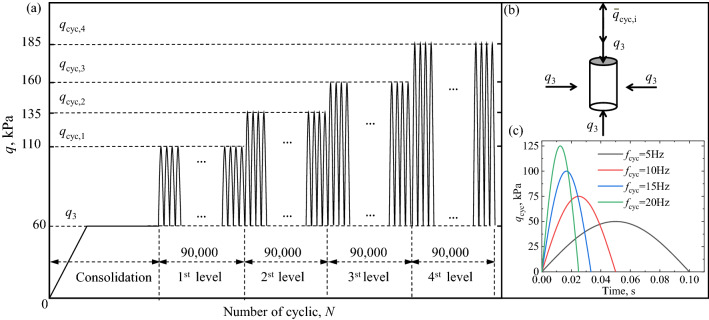
Loading patterns: (**a**) focusing on *q*_cyc_, (**b**) schematic diagram of stress path on sample and (**c**) focusing on *f*_cyc_.

Figure 3 shows the loading patterns in cyclic triaxial tests. Table 2 lists the details of the experimental program. Characteristic frequencies (*f*_c_) have a significant influence on the modulation amplitude of embankment vibration. The *f*_c_ was calculated by considering the train speed and the distance of the wheel base^43^. Therefore, four different *f*_cyc_ (5 Hz, 10 Hz, 15 Hz, and 20 Hz) were considered to simulate the *f*_c_ caused by high-speed train operation. The lateral confinement (*q*_3_) provided by the track board, Cement Asphalt (CA) mortar, concrete base, and subgrade bed was mainly in the vicinity of 60 kPa, as indicated by the Tian^14^. The *q*_cyc_ was increased from 50 to 125 kPa, in increments of 25 kPa^14^. For each *q*_cyc_, 90,000 loading cycles were applied. This was considered sufficient for stabilizing the permanent deformation at the ending cycles of a given stress level^28–30,44^. The number of load applications is 360,000, which reflect the long-term cyclic loading.

**Table 2:** Summary of cyclic triaxial tests.

Test name	Cycle loading frequency, *f*_cyc_ (Hz)	Freezing–thawing cycle, *N*_FT_	Fines matrix content, FC (%)	Confining pressure, *q*_3_ (kPa)	Deviator stress amplitude, *q*_cyc_ (kPa)	Number of load applications, *N*
UFT1/FT2	20	0/10	21	60	50/75/100/125	90,000 × 4 = 360,000
FT3/FT4/FT5	5/10/15	10	21			
FT6/FT7/FT8	20	10	6/11/16			

### Resilient modulus results

**Figure 4:**
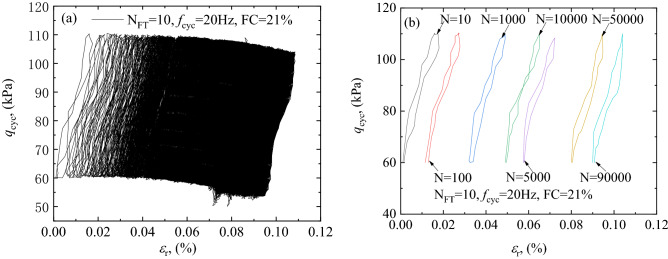
Hysteresis loop development characteristics: (**a**) hysteresis cycle of the sample under 90,000 loads, (**b**) hysteresis cycle of the sample under different loads.

The test results named FT2 is selected to show the hysteresis behavior, as shown in Fig. 4. The shape and slope of hysteresis loop reflect the damping of sample and modulus of sample.

**Figure 5:**
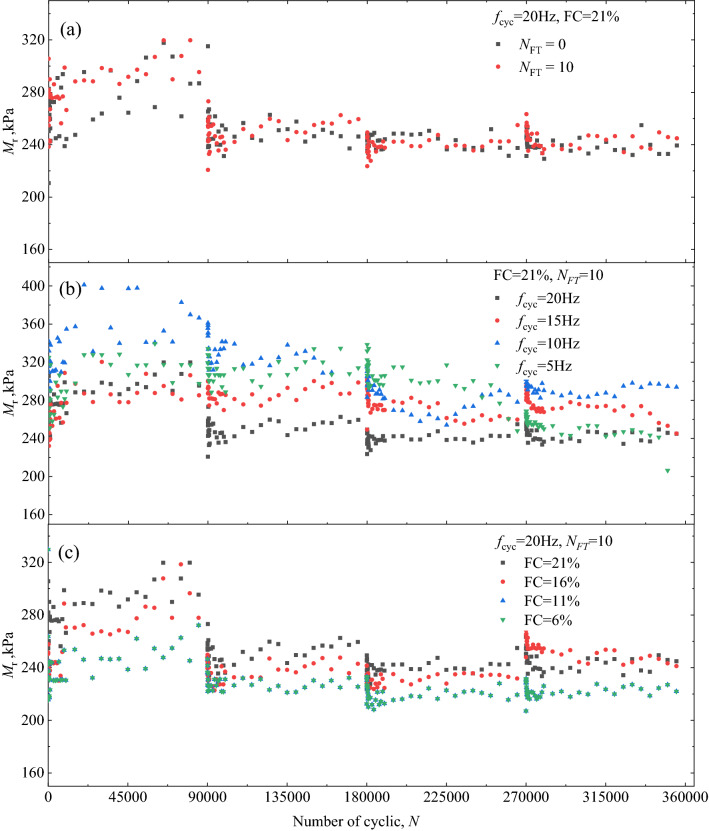
Relationship between *M*_r_ and number of load applications: (**a**) focusing on the effect of *N*_FT_; (**b**) focusing on *f*_cyc_; (**c**) focusing on FCFreezing–thawing cyclic.

Figure 5 presents the relationship between *M*_r_ and number of load applications (*N*). The calculation of *M*_r_ were displayed in author’s early work^15^. The effect of *N*_FT_, *f*_cyc_ and FC on *M*_r_ were shown in Fig. 5a–c respectively.

**Figure 6:**
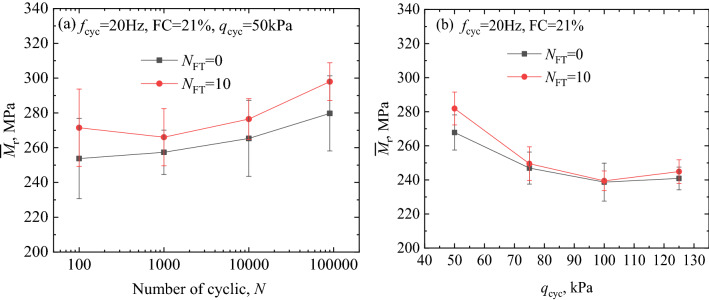
Relationship between ${\overline{M}}_{\text{r}}$ and *N*, *q*_cyc_: focusing on *N*_FT_: (**a**) focusing on the effect of *N* and (**b**) focusing on *q*_cyc_.

**Figure 7:**
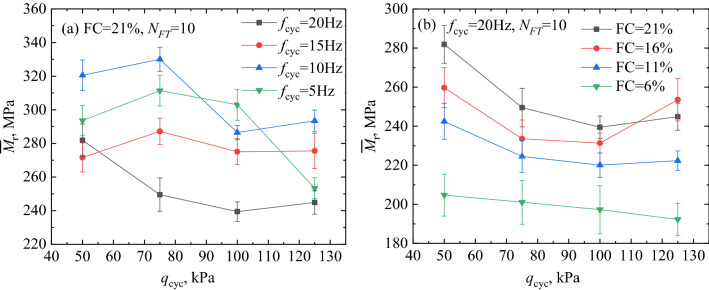
Relationship between ${\overline{M} }_{\text{r}}$ and *q*_cyc_, (**a**) focusing on *f*_cyc_ and (**b**) focusing on FC.

Certain fluctuations were observed in the results of the *M*_r_ in the long-term load applications. The mean value of *M*_r_ was denoted as ${\overline{M}}_{\text{r}}$ (${\overline{M}}_{\text{r}}\text{=}\left(\sum_{{i}}^{{n}}{{\text{M}}}_{\text{ri}}\right)/\left(n-{\text{i}}\right)$). The ${\overline{M}}_{\text{r}}$ and standard deviation of resilient modulus were calculated for subsequent analysis. The *M*_r_ value from the 50 to 150 times, 500 to 1500 times, 5000 to 10,000 times and 80,000 to 90,000 times were averaged and shown in Fig. 6a. In order to analysis the effect of *q*_cyc_, *f*_cyc_ and FC on *M*_r_, the mean value of *M*_r_ under each *q*_cyc_ is calculated and shown in Figs. 6b, 7a,b.

Figures 5b and 6a show the variation in the ${\overline{M}}_{\text{r}}$ of the specimens under cyclic loading at a frequency of 20 Hz. After the freezing–thawing cycles of 10 (*N*_FT_ = 10), the ${\overline{M}}_{\text{r}}$ of specimen was larger than that of the specimen that did undergo freezing–thawing cyclic, thus indicating that the freezing–thawing effect promoted the resistance to deformation of the coarse and fine matrix mixture geomaterials. With an increase in the number of cycles of cyclic loading (*N*), the ${\overline{M}}_{\text{r}}$ of the specimens gradually increased (as shown in Fig. 6a). This was because the specimen underwent the cyclic compaction process at the initial stage of loading, which was similar to the results obtained in previous studies^25^. Additionally, with an increase in *q*_cyc_, the ${\overline{M}}_{\text{r}}$ of the specimens decreased significantly, thus indicating that the increase in *q*_cyc_ degraded the strength of the coarse and fine mixture geomaterials, as shown in Fig. 5b.

### Cyclic loading frequency

Figure 7a shows the variation in the ${\overline{M}}_{\text{r}}$ of the specimens with a fine matrix content of 21%, after 10 freezing–thawing cycles. Although the increase in *f*_cyc_ (from 5 to 10 Hz) improved the strength of the specimens, when the loading frequency exceeded 10 Hz, the ${\overline{M}}_{\text{r}}$ of the specimens decreased with increasing of *f*_cyc_ (from 10 to 20 Hz). Compared with the loading frequency of 20 Hz, the *q*_cyc_ has a certain effect on the strength of the specimens at low *f*_cyc_ (from 5 to 15 Hz), which is reflected in that the ${\overline{M}}_{\text{r}}$ of the specimens increases with the increase of the *q*_cyc_ (from 50 to 75 kPa). Furthermore, the strength degradation of the specimen gradually appeared in the third stress amplitude (*q*_cyc_ = 100 kPa). Low *f*_cyc_ under low *q*_cyc_ promoted the compactness of the specimen. Whereas, high *f*_cyc_ under high *q*_cyc_ significantly degraded the strength of the specimen. In addition, the loading frequency duration of 5 Hz was 4 times that of 20 Hz under the same number of cycles. Therefore, the fatigue effect on the specimen was reflected in the fourth stage of loading (*q*_cyc_ = 125 kPa), where the strength deteriorated rapidly.

### Fine matrix content

Figure 7b shows the variation in the ${\overline{M}}_{\text{r}}$ of the specimens having different FC (from 6 to 21%), after 10 freezing–thawing cycles under dynamic loading, at a cyclic loading frequency of 20 Hz. The ${\overline{M}}_{\text{r}}$ increased with the increase in FC indicating that the fine matrix clearly improved the strength of the specimen. The fine matrix played a significant role in filling the pores of the specimens dominated by coarse particles, which resulted in the specimen having good compactness. Additionally, with an increase in *q*_cyc_ and *N*, the strength of the specimen deteriorated. However, the strength of the specimens with higher fine matrix content slightly increased under long-term cyclic loading. This behavior was similar to that of the coarse and fine mixed interlayer formed in French railways, which indicated that the relative size of the coarse and fine matrix in the coarse and fine mixed filler has a crucial influence on the strength of the filler^7,8^.

## Discussion

### Freezing–thawing effects

There are two different results about the influence of freeze–thaw cycles on ${\overline{M}}_{\text{r}}$ of coarse–fine mixtures as mentioned in introduction part. The result of present work is consistent with previous work^17,18^. To the best of our knowledge, both the rate of freezing–thawing cycle and particle composition must be considered as key factors contributing to the differences in the aforementioned research results.

**Figure 8:**
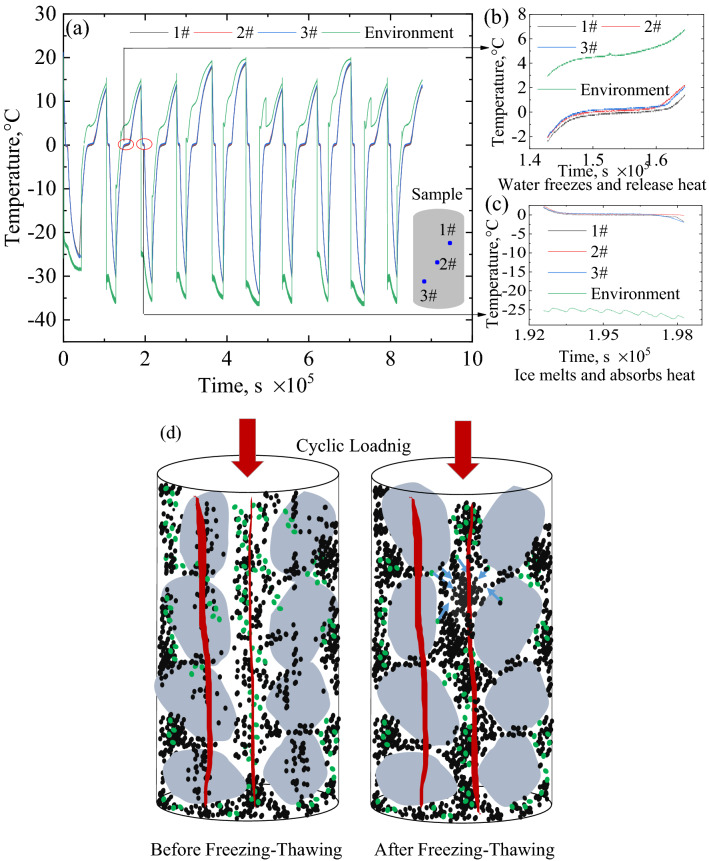
The process of fine matrix clusters under freezing–thawing cyclic and cyclic loading: (**a**) focusing on the temperature change during freezing–thawing, (**b**) detail diagram of Release the latent heat of the phase transition, (**c**) detail diagram of absorb the latent heat of the phase transition and (**d**) schematic diagram of fine matrix clusters filling pores before and after freezing and thawin.g Additionally, the coarse particles used in present test were unweathered granite, and there was no evident initial crack on its surface. Therefore, the coarse particles were not damaged during the freezing–thawing cycle. X-ray diffraction (XRD) test results of the fine matrix of the specimen indicated that it contained a large amount of montmorillonite, albite and minerals which possessed strong water–holding capacity, as shown in Fig. 1b. (**d**) Demonstrate the process that liquid water and fine matrix on the surface of coarse particles will be absorbed by the cluster body in the process of repeated freezing and thawing. The cluster rate increased by the migration of fine particle matrix driven by temperature load during repeated freeze–thawing process. For the fine particle matrix with strong water holding capacity, the clustering phenomenon should be more obvious during freeze–thaw process. Previous research has supported above conjecture to some extent^39,49^. The pores formed by the coarse and fine matrix skeleton were filled with the cluster body, which improved the resistance to deformation of the specimen. Furthermore, under the condition of a low content of fine matrix, liquid water was discharged from the bottom of the specimen after 10 freezing–thawing cycles. This was consistent with the work from previous work, where in the liquid water discharge undoubtedly enhanced the strength of the specimen^19^. However, the effect of the liquid water discharge on the strength of the specimen was much less evident than that of fine matrix clusters filling the pores. The dynamic loads of the specimens were borne by the coarse–particle skeleton and clusters. (**b**) Shows the schematic diagram of the force chain inside the specimen. Therefore, at present work, specimens with a higher fine matrix exhibited higher strength after freezing–thawing than those with a lower fine matrix.

In the present study, the temperature of the specimens throughout the entire freezing–thawing cycle was continuously tested, as shown in Fig. 8a. More specifically, the phenomenon of exothermic and endothermic transformation in the freezing–thawing process of the sample is also captured, as shown in Fig. 8b,c. The freezing and thawing times of the specimen were both 6 h. Specifically, the freezing and thawing rates were 10 ℃/h and 8 ℃/h, respectively. Table 3 lists the data of the freezing–thawing cycle temperatures from previous studies^15,45–48^. We observed that the specimens in the present test underwent a rapid freezing–thawing cycle. In the process of rapid freezing and thawing, there is a negligible difference in temperature gradient inside the specimen. Hence the physical process of water migration does not have sufficient time to occur. The state of the specimen is uniform and stable during the freezing–thawing cycle. This was one of the important reasons for the present test being different from previous studies, particularly, the rapid freezing–thawing cycle rate did not degrade the strength of the coarse and fine matrix mixture.

**Table 3:** Summary of freezing and thawing temperature and holding time from previous work.

References	Freezing temperature (℃)	Freezing time (hour)	Thawing temperature (℃)	Thawing time (h)
Oztas^45^	− 4 and − 18	24	5	24
Wang^46^	− 15	12	5	12
Tan^47^	− 16	12	25	12
Chang^48^	− 5	12	20	12
Tian^15^	− 20	12	20	12
Present study	− 35	6	15	6

### Kinetic effects of rate dependent

**Figure 9:**
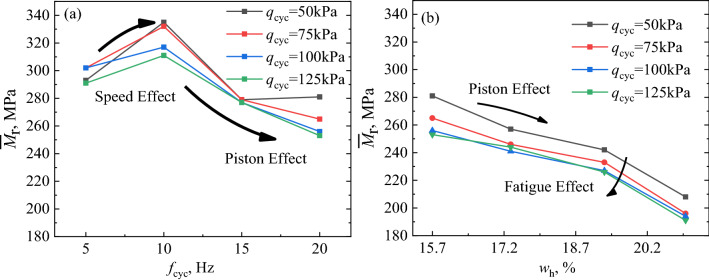
Evolution of kinetic effect based on the experimental data: (**a**) focusing on the effect of *f*_cyc_ on ${\overline{M} }_{\text{r}}$, (**b**) focusing on the effect of *w*_h_.

The strength variation rule of FTCFG under the influence of freeze–thaw cycle is deeply analyzed. Three kinetic effects of FTCFG materials under the influence of *f*_cyc_ will be discussed in the next section. In Fig. 9, the ordinate is the ${\overline{M}}_{\text{r}}$ and the abscissa includes the *f*_cyc_ and the *w*_h_. Three typical kinetic effects of FTCFG, namely rate effect, fatigue effect, and piston effect, were observed under the effect of *f*_cyc_. When the *f*_cyc_ increased from 5 to 10 Hz, the specimen clearly exhibited the rate effect. The ${\overline{M}}_{\text{r}}$ increased by 14.3%, 9.9%, 4.9%, and 6.8% under the different *q*_cyc_, respectively, and this was consistent with previous work^19^. The rate effect is related to the particle composition and deformation mechanism^50^. A certain physical and chemical connection was observed at the contact point of the specimen formed by coarse and fine mixture geomaterials. However, the weak connection resulted in a constant adjustment of the specimen particle arrangement under dynamic cyclic loading^50^. When the loading frequency increased in the range below 10 Hz, the adjustment of particle arrangement could not be fully completed in the short time, and the deformation was not fully developed. Therefore, the ${\overline{M}}_{\text{r}}$ of the specimens increased with the increase in loading frequency. Additionally, the test results revealed the fatigue effect on the specimens under long-term dynamic load. The coarse particles were broken under long-term loading, which led to the destruction of the original stable skeleton and pore structure of the geomaterials, and the ${\overline{M}}_{\text{r}}$ of the specimen continued to decrease^50^.

The ${\overline{M}}_{\text{r}}$ of the specimens decreased rapidly with the increase in *f*_cyc_ (10 Hz ≤ *f*_cyc_ ≤ 20 Hz). Moreover, the geomaterials with particle size less than 2 mm demonstrated water holding capacity. The *w*_h_ of the specimens with different FC (21%, 16%, 11%, and 6%) were calculated to be 15.7%, 17.35%, 19.31%, and 21.01%, respectively, as shown in Table 1. Analogously, the ${\overline{M}}_{\text{r}}$ of the specimens having lower fine matrix content clearly decreased with the increase in *w*_h_. The phenomenon of the ${\overline{M}}_{\text{r}}$ decreasing with the increase in *f*_cyc_ and *w*_h_ was described as the piston effect. Under high-frequency cyclic loading, excessive pore pressure was generated in the specimen. In this situation, if the saturation of the specimen is higher, the excess pore pressure in the specimen is more difficult to dissipate^51^. The existence of excess pore pressure leads to the decrease in ${\overline{M}}_{\text{r}}$ of the specimen, and the same phenomenon appears in the study of saturated sand^52^. In this study, the threshold value of the loading frequency that caused the piston effect in the FTCFG was 10 Hz. The test results showed that the effect of the vibration piston on the deterioration of the specimen strength was stronger than that of the rate effect on the improvement of the specimen strength.

**Figure 10:**
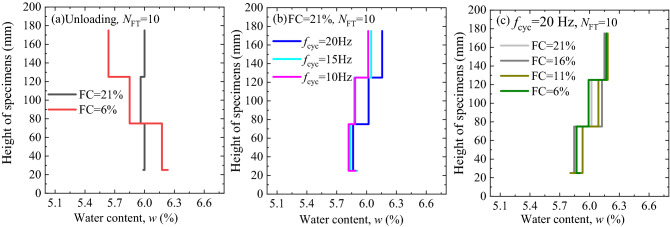
Variations in specimen height with *w* in different work conditions (**a**) focusing on freezing–thawing cyclic; (**b**) focusing on FC; (**c**) focusing on *f*_cyc_.

Furthermore, the piston effect could cause the capillary water at the bottom of the specimen to migrate upwards. The height of the specimen (H) is 200 mm and it is divided into 4 equal parts from top to bottom. The water content (*w*) of each part was measured by drying method. Water content distribution of specimens under different test conditions is shown in Fig. 10. After 10 freezing–thawing cycles, the specimen with a less fine matrix exhibited evidently poor water-holding characteristics, which led to the water in the specimen accumulating at the bottom of the specimen due to gravity stress. Contrarily, the specimen with 21% fine matrix content did not demonstrate evident water field redistribution after 10 freezing–thawing cycles, as shown in Fig. 10a. Additionally, for the specimen with 21% fine matrix content, the water content at the top of the specimen increased, compared with that at the bottom, after cyclic loading, as shown in Fig. 10b. Similarly, for the specimen with a loading frequency of 20 Hz, the water content at the top of the specimen with different fine matrix contents was higher than that at the bottom, after cyclic loading, as shown in Fig. 10c. Therefore, the results indicated that the piston effect had a significant effect on the water redistribution in the specimens. These conclusion are of great significance to practical engineering.

### Resilient modulus rate dependent model

Numerous efforts have been devoted to establish the relationship between *M*_r_ and stress level. One of the most widely used formulations was proposed by Uzan^34^ as follows:

1 $${M}_{r}=a{\left(\frac{{q}_{1}+{q}_{2}+{q}_{3}}{{p}_{atm}}\right)}^{b}{\left(\frac{{q}_{cyc}}{{p}_{atm}}\right)}^{c},$$

where ${q}_{1}$, ${q}_{2}$ and ${q}_{3}$ are principal stresses; *q*_cyc_ is deviator stress amplitude;${p}_{atm}$ is atmospheric pressure; a, b and c are regression coefficients.

Clearly, Eq. (1) only can descript the effect of initial volume stress and *q*_cyc_ on ${M}_{r}$, as it ignores geomaterials suction, especially for unsaturated geomaterials. A new equation is proposed by Ng^36^ to descript the effect of geomaterials suction as follows:

2 $${M}_{r}={M}_{0}{\left(\frac{q}{{p}_{atm}}\right)}^{{k}_{1}}{\left(\frac{{q}_{cyc}}{{p}_{atm}}\right)}^{{k}_{2}}{\left(1+\frac{s}{p}\right)}^{{k}_{3}},$$

where net mean stress, *q*, is defined as $\left[\frac{\left({q}_{1}+{q}_{2}+{q}_{3}\right)}{3}-{\mu }_{\text{a}}\right]$; *s* is matric suction (${\mu }_{\text{a}}$ − ${\mu }_{\text{w}}$), ${\mu }_{\text{a}}$ and ${\mu }_{\text{w}}$ are pore air pressure and pore water pressure, respectively; $p$ = ${p}_{atm}$;${M}_{0}$ ${k}_{1}$, ${k}_{2}$ and ${k}_{3}$ are regression exponents.

Equation (2) allows for a smooth transition between an unsaturated geomaterials and a saturated geomaterials. When matric suction is zero, the fourth term reduces to 1.0. Then this equation can be applied to determine *M*_r_ of a saturated geomaterials from effective confining pressure and cyclic stress.

However, when *q*_cyc_ approaches 0 (i.e., at very small strains), a very large *M*_r_ can be predicted because the exponent *k*_2_ is negative. The second term on the right–hand side of Eq. (3) is optimized to more accurately evaluate the ${M}_{r}$ under the condition of small stress amplitude. The limitation of Eq. (2) can be simply overcome by replacing the term $\left(\frac{{q}_{cyc}}{{p}_{atm}}\right)$ by $\left(1+\frac{{q}_{cyc}}{{p}_{atm}}\right)$. Then, this equation can be rewritten as Ng^35^:

3 $${M}_{r}={M}_{0}{\left(\frac{q}{{p}_{atm}}\right)}^{{k}_{1}}{\left(1+\frac{{q}_{cyc}}{{p}_{atm}}\right)}^{{k}_{2}}{\left(1+\frac{s}{p}\right)}^{{k}_{3}}.$$

According to explore and discuss the experiment results systematically, the influence factors of ${M}_{r}$ of the FTCTG include ${f}_{cyc}$ and ${w}_{h}$ except stress level ($p,{q}_{cyc}$). Hence, based on the Eq. (3), a long-term resilient modulus rate dependent model was developed which can consider the complexity of the nonlinear influence process of ${f}_{cyc}$ and *q*_cyc_ on ${M}_{r}$ and the advantage of ${w}_{h}$ in representing the state of FTCTG. Net mean stress, dynamic stress, cyclic Loading frequency and material compositions are simultaneously considered in the new rate-dependent model as shown in Eq. (4). The difference between the model proposed in this manuscript and the previous models is that the former considers the influence of dynamic loading frequency change on the ${M}_{r}$ of materials. Furthermore, there are also difference for the response of different loading frequency on different material compositions, which is also affected by different dynamic stress amplitudes. More details about Eq. (4) will be displayed in the next section.

4 $${M}_{r}=f\left(p,{q}_{cyc}\right)\cdot g\left({f}_{cyc}\right)\cdot h\left({w}_{h}\right),$$

where $f\left(p,{q}_{cyc}\right)$ is the equation to express two stress state variables; $g\left({f}_{cyc}\right)$ is the equation to express the effect of ${f}_{cyc}$ on ${M}_{r}$; $h\left({w}_{h}\right)$ is the equation to express the effect of water–holding capacity on ${M}_{r}$.

### Dynamic stress level correction function

The first term on the right-hand side of Eq. (4) is systematically improved to evaluate the effect of *q*_cyc_ at different loading frequencies on ${M}_{r}$ of FTCFG. Both net mean stress and dynamic stress are included in Eq. (5), which related to initial volume stress and deviator stress amplitude respectively.

5 $$f\left(p,{q}_{cyc}\right)={M}_{0}{\left(\frac{p}{{p}_{atm}}\right)}^{{k}_{1}}{\left(1+\frac{{q}_{cyc}}{{p}_{atm}}\right)}^{{k}_{2}}.$$

The first term on the right–hand side of Eq. (5) denotes the equation of net mean stress. ${M}_{r}$ is proportional to the net mean stress at Cam–clay model^36^. In addition, previous work investigated the relationship between G_0_ and net mean stress, and the results show that the value of ${k}_{1}$ is between 0.75 and 1^53^. Therefore, To simplify the model parameters, where ${k}_{1}=1$, ${p}_{atm}=1 \,\text{kPa}$, and $p=60\, \text{kPa}$.

Then, the second term on the right-hand side of Eq. (5) quantifies the influence of ${q}_{cyc}$ on ${M}_{r}$. According to the experiment results and discussion, the second term needs to be optimized to consider the effect of ${f}_{cyc}$ and loading history on ${M}_{r}$ especially, for long-term cyclic loading application. When ${f}_{cyc}$ is more than threshold of cyclic loading frequency (${f}_{\text{T}}$), the increase of ${q}_{cyc}$ deteriorates the ${M}_{r}$ of FTCFG significantly. Therefore, the second term on the right-hand side of Eq. (5) can be used to describe the relationship between *q*_cyc_ and ${M}_{r}$, as shown in Eq. (6).

6 $$f\left({q}_{cyc}\right)={\left(1+\frac{{q}_{cyc}}{{p}_{atm}}\right)}^{{k}_{2}},$$

where ${k}_{2}$ is regression exponent, also the exponent ${k}_{2}$ is negative because ${M}_{r}$ decreases with increasing *q*_cyc_.

When ${f}_{cyc}$ is not more than ${f}_{\text{T}}$, the increase of ${q}_{cyc}$ has a certain improvement effect on ${M}_{r}$. Since the ${M}_{r}$ does not increase monotonically with the *q*_cyc_, the amplitude–frequency coordination coefficient (${k}_{2p}$ and ${k}_{2n}$) and referenced deviator stress amplitude (${q}_{ref}$) are introduced to characterize the strengthening and deterioration effect of the increase of *q*_cyc_ on the ${M}_{r}$. Therefore, the second term on the right–hand side of Eq. (5) can be rewritten as follows:

7 $$f\left({q}_{cyc}\right)={\left(1+\frac{{q}_{cyc}}{{p}_{atm}}\right)}^{{k}_{2p}}{ q}_{cyc}\le {q}_{ref},$$

8 $$f\left({q}_{cyc}\right)={\left(1+\frac{\left({q}_{cyc}-{q}_{ref}\right)}{{p}_{atm}}\right)}^{{k}_{2n}}+f\left({q}_{ini}\right){ q}_{cyc}\ge {q}_{ref},$$

where amplitude–frequency coordination coefficient, ${k}_{2p}$ should be positive because ${M}_{r}$ increase with increasing ${q}_{cyc}$ in Eq. (7). Also, ${k}_{2n}$ should be negative because ${M}_{r}$ decrease with increasing ${q}_{cyc}$ in Eq. (8); where this term, (${q}_{cyc}$–${q}_{ref}$), represents the elimination of loading history in Eq. (8); where $f\left({q}_{ini}\right)$ considers the cumulative effect of low amplitude loading, which is related to ${q}_{ini}$, as shown in Eq. (9).

9 $$f\left({q}_{ini}\right)={(1+{q}_{ini})}^{d},$$

where *d* is regression exponents; the exponent *d* is negative because the cumulative strengthening effect increase with the ${q}_{ini}$ of decrease.

**Figure 11:**
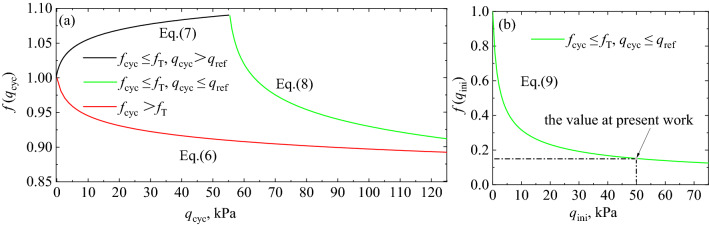
Dynamic stress level correction function: (**a**) focusing on Eqs. (6), (7), and (8); (**b**) focusing on Eq. (9).

The above equation can be illustrated in Fig. 11, which shown the effect of ${q}_{cyc}$ on the ${M}_{r}$ of FTCFG.

### Cyclic loading frequency correction function

The second term on the right-hand side of Eq. (4) is deduced to evaluate the effect of ${f}_{cyc}$ on ${M}_{r}$ of FTCFG. As previously discussed, there are two different effects on the ${M}_{r}$ of FTCFG under long-term cyclic loading. The reference frequency (${f}_{ref}$) was introduced to characterize the behaviours of resilient modulus rate dependent under cyclic loading application. The equation of rate dependent be proposed as:

10 $$g\left({f}_{cyc}\right)=1.1{e}^{\frac{{-\left({f}_{cyc}-{f}_{ref}\right)}^{2}}{2\times {f}_{cor}^{2}}},$$

where ${f}_{cor}$ is resilient modulus affects coefficient, which determines the degree of influence of long-term cyclic loading on ${M}_{r}$ of FTCFG; ${f}_{ref}$ is reference frequency; Based on results of present work, the ${f}_{ref}$ is 10 Hz.

**Figure 12:**
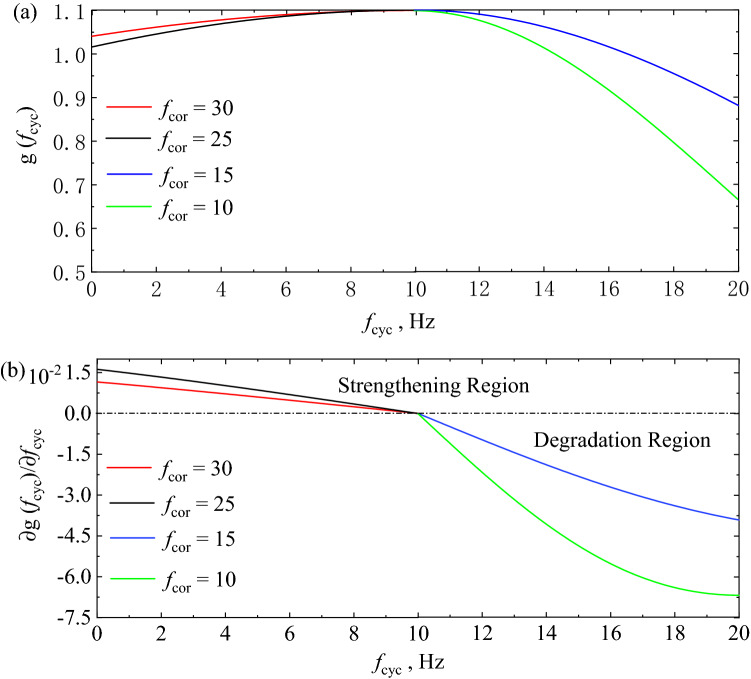
Cyclic loading frequency correction function, (**a**) focusing on *f*_cyc_ and (**b**) enhancement and degradation degree of ${M}_{r}$ effected by *f*_cyc_.

Figure 12a illustrates the modified result with different values of ${f}_{cor}$. When ${f}_{cyc}$ is not more than ${f}_{\text{ref}}$, the cyclic loading application is not enough to cause the increase of pore pressure of FTCFG. Therefore, due to the rate effect caused by long-term cyclic loading, the ${M}_{r}$ of FTCFG has a certain increase, and the equation of rate dependent shows a monotonic increasing trend. When ${f}_{cyc}$ is more than ${f}_{\text{ref}}$, the deterioration effect of the increase of pore pressure which caused by the long-term high frequency cyclic loading on the modulus has exceeded the strengthening effect which caused by the rate effect, and the equation of rate dependent shows a monotonic decreasing trend.

Furthermore, Eq. (11) is obtained by differentiating *f*_cyc_ in Eq. (10).

11 $$\frac{\partial g({f}_{cyc})}{\partial {f}_{cyc}}=\frac{1.1{e}^{\frac{-{\left({f}_{cyc}-{f}_{ref}\right)}^{2}}{2\times {f}_{cor}^{2}}}({f}_{cyc}-{f}_{ref})}{{f}_{cor}^{2}},$$

where parameters in Eq. (11) are the same as Eq. (10).

Figure 12b illustrates the result of Eq. (11) with different values of ${f}_{cor}$, which can be used to characterize the influence of different *f*_cyc_ on ${M}_{r}$ of FTCFG. The positive value indicates the strengthening effect of long-term cyclic loading on ${M}_{r}$ of FTCFG, and the negative value indicates the deterioration effect of long-term cyclic loading on ${M}_{r}$ of FTCFG. The influence of long-term cyclic loading on ${M}_{r}$ of FTCFG is reflected in the superposition of strengthening and deterioration effects. The strengthening effect of long-term cyclic loading on ${M}_{r}$ of FTCFG always exists, but the deterioration effect caused by the increase of frequency exceeds the strengthening effect, and finally shows the deterioration effect.

### Moisture holding capacity correction function

The effect of matric suction on ${M}_{r}$ of unsaturated coarse and fine particle mixture was expressed in the fourth term on the right-hand side of Eq. (3)^36^. To the best of our knowledge, the influence of matric suction on ${M}_{r}$ depends on water content of FTCFG. The drying curve and wetting curve in soil–water characteristic curve (SWCC) constitute a hysteretic cycle^54^. Since that the geomaterials with the same water content has two different matric suction values in SWCC, the application of Eq. (3) is controversial. In addition, since that water content is easier to obtain than matric suction for geomaterial, water content is used to quantify the effect of geomaterial state on ${M}_{r}$. Furthermore, considering the fact that coarse particles in FTCFG (particle size more than 2 mm) do not hold water, if mass or volume water content of FTCFG is directly used to evaluate the ${M}_{r}$ of FTCFG, it will undoubtedly be overrated. Therefore, the water-holding moisture content (${w}_{h}$) is introduced to characterize the influence of material state on its ${M}_{r}$. The ${w}_{h}$ is defined as the ratio of the mass of water to the mass of fine particle (particle size less than 2 mm). A new parameter, ${w}_{h}$, is used to evaluate the variation of ${M}_{r}$ of FTCFG instead of matrix suction, which is not only more valuable in engineering application but more intuitive in reflecting the state of FTCFG.

Therefore, the third term on the right-hand side of Eq. (4) is developed to characterize the effect of water-holding capacity on ${M}_{r}$ of FTCFG. Combined with SWCC, the equation considering the variation of water holding capacity can be proposed as:

12 $$h\left({w}_{h}\right)={e}^{\frac{{-\left({w}_{h}-{w}_{opt}\right)}^{2}}{2\times {w}_{liq}^{2}}},$$

where ${w}_{opt}$ is the optimum water content which depends on the grain composition; ${w}_{liq}$ is liquid limit of fine particle.

**Figure 13:**
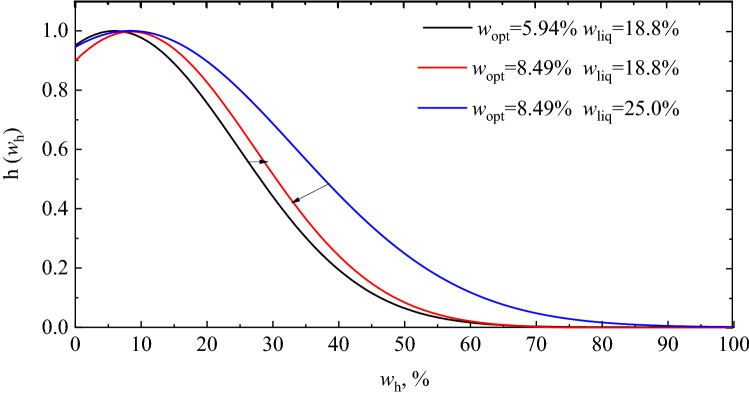
Moisture holding capacity correction function.

Figure 13 illustrates the result of Eq. (12). When the ${w}_{h}$ of FTCFG is less than the ${w}_{opt}$, the ${M}_{r}$ of FTCFG increases with the increase of ${w}_{h}$ under cyclic loading application. On the contrary, with the increase of ${w}_{h}$, the ${M}_{r}$ of FTCFG decreases rapidly under cyclic loading. ${w}_{opt}$ in Eq. (12) is the threshold value that determines the influence of ${M}_{r}$ of FTCFG to be strengthened or deteriorated. ${w}_{liq}$ in Eq. (12) can be used to characterize the deterioration rate of ${M}_{r}$ of FTCFG. When the geomaterials reaches saturation, its strength is almost lost. Considering that the ${w}_{h}$ of the sample in this study is greater than the ${w}_{opt}$, the ${M}_{r}$ of FTCFG gradually deteriorates with the increase of ${w}_{h}$.

### Verification of the proposed modeling

**Figure 14:**
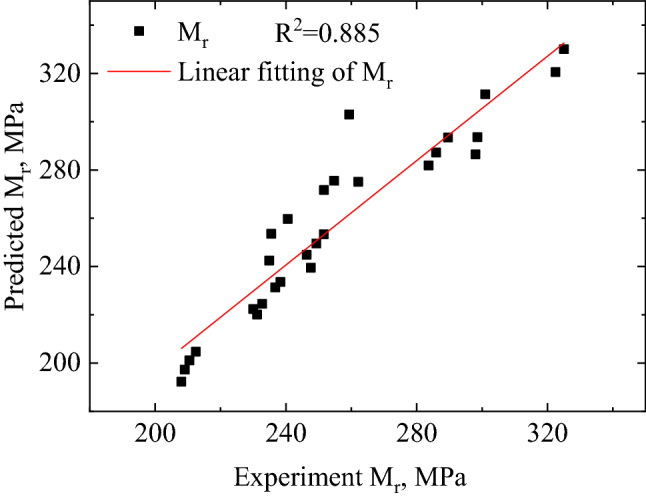
Predicted versus measured resilient modulus.

Figure 14 compares the predicted ${M}_{r}$ of FTCFG using Eq. (4) with experimental ${M}_{r}$ of FTCFG from the laboratory tests. The data sources for determining the parameters of model (Eq. (4)) are experimental test those test name are UFT1, FT2, FT3 and FT4. The data sources for validation of model (Eq. (4)) are experimental test those test name are FT5, FT6, FT7 and FT8. A coefficient of determination (R^2^) of 0.885 indicated a good correlation between predicted and measured results for the FTCFG. The parameter values are summarized in Table 4.

**Table 4:** Summary of regression coefficients of proposed resilient modulus rate dependent model.

Equations	Parameters	Values	Unit
Equation (5)	${M}_{0}$	4.6	–
	$p$	60	kPa
	${p}_{atm}$	1	kPa
	${k}_{1}$	1	–
Equation (6)	${k}_{2}$	− 0.0235	–
Equation (7)	${k}_{2p}$	0.02	–
Equation (8)	${k}_{2n}$	− 0.05	–
	${q}_{ref}$	50	kPa
Equation (9)	*d*	− 0.48	–
Equation (10)	${f}_{ref}$	10	Hz
	${f}_{cor}$	10, 15, 25, 30	–
Equation (12)	${w}_{liq}$	18.8	%
	${w}_{opt}$	Table 1	–

## Conclusion

A long-term resilient modulus rate dependent model for freezing–thawing coarse–fine mixtures geomaterials was developed by conducting the cyclic triaxial test which could aid in understanding the effects of cyclic loading frequency on the *M*_r_ of FTCFG. The following conclusions were drawn.

The strengthening effect of *M*_r_ of geomaterials after freezing–thawing cyclic was explored by the cyclic triaxial test. There are two reason could explain why the *M*_r_ of geomaterials is increase underwent the freezing–thawing cyclic. Rapid freezing–thawing can improve the fine matrix cluster rate and fill the coarse particle pores. Coarse particles in FTCFG do not hold water that results in the discharge of liquid water.

The three kinetic effect of the influence of *f*_cyc_ on *M*_r_ of FTCFG was revealed by the discussion of experiment results. The rate effect of the geomaterial comes into play and intensifies the *M*_r_ of FTCFG, when the *f*_cyc_ lower than 10 Hz. The piston effect of the geomaterial comes into play and degrades the *M*_r_ of FTCFG, when the *f*_cyc_ more than 10 Hz. In addition, the degradation of *M*_r_ of FTCFG by piston effect is more obvious in geomaterial with high ${w}_{h}$. Fatigue effect gradually shows deterioration of *M*_r_ of FTCFG after long-term cyclic loading application.

A long-term resilient modulus rate dependent model describing the effect of *q*_cyc_, *f*_cyc_ and *w*_h_ on *M*_r_ of FTCFG is proposed. The effect of *q*_cyc_ and *f*_cyc_ on *M*_r_ of FTCFG is coordinate with each other. Using ${w}_{h}$ to characterize the influence of geomaterial state change on *M*_r_ is not only of clear physical significance but also beneficial to engineering application. This new model is able to capture the variation of *M*_r_ of FTCFG with both dynamic stress level and saturation of geomaterial. For the four FTCFG with different FC verified, the *M*_r_ of FTCFG values predicted using this equation are generally consistent with the measured results.

## Data Availability

The datasets generated and/or analysed during the current study are not publicly available due [This study was financially supported by the National Natural Science Foundation of China (Grant No. 42102311), which is currently under study] but are available from the corresponding author on reasonable request.
